# Metagenomic Exploration of Microbial Signatures on Periyar River Sediments from the Periyar Tiger Reserve in the Western Ghats

**DOI:** 10.1128/genomeA.00154-18

**Published:** 2018-03-15

**Authors:** Aparna Chakkamadathil Rajeev, Nishi Sahu, Maushumi Deori, Suma Arun Dev, Vijay Pal Yadav, Ilora Ghosh

**Affiliations:** a School of Environmental Sciences, Jawaharlal Nehru University, New Delhi, India; b Division of Forest Genetics and Biotechnology, Kerala, Forest Research Institute, Kerala, India

## Abstract

We report here targeted deep-sequencing metagenomic data that reveal a high level of diversity in the microbiota residing in the sediment of the Periyar River in a reserve forest of the Western Ghats. Of the 4,674 operational taxonomic units discovered, the dominant phyla represented were Proteobacteria (33.12%), Actinobacteria (14.58%), Acidobacteria (12.81%), and Bacteroidetes (9.89%).

## GENOME ANNOUNCEMENT

The Western Ghats is one among the eight megadiversity hot spots in the world, along with the Indo-Burma region from India, owing to its exceptionally high level of biological diversity and endemism. Covering an area of only 182,500 km^2^ , it harbors around 30% of all faunal and floral diversity in India (1).

Although we continue to learn more about the floral (2) and faunal (3, 4) diversity of the Western Ghats, little is currently known about its microfloral diversity. Culture-based methods and bioprospecting studies using metagenomic clonal libraries have resulted in the discovery of some unique bacterial species and genes, but these have not been powerful enough to capture the full taxonomic diversity of the region (5, 6). A vast bulk of the microbes are unculturable in standard laboratory media, and their signatures are absent from most of the taxonomic data. With the advent of next-generation sequencing technology, we can tap into these unexplored niches (7). Metagenomic sequencing studies on the soils of the Indo-Burma biodiversity hot spot (8) and the earth microbiome project (9) are slowly closing this lacuna of information.

The Periyar River, the longest perennial river in the Western Ghats, is popularly called “the lifeline” of the Indian State of Kerala. It stretches 300 km, passing through urban and industrial zones to drain into the Vembanad Kol wetlands (Ramsar Site), and it is cited as one of the most polluted river systems in India (10). Here, we report on the microbiota of Periyar River sediment samples obtained from where the river is in pristine form, that is, at its origin, which is located within the Periyar Tiger Reserve (9.575385°N, 77.179488°E), during the premonsoon season (March 2017). Samples were kept at 4°C in the field and later stored at −20°C until they could be further analyzed. Representative samples were subsequently processed for DNA extraction using a DNeasy PowerSoil kit (Qiagen, Inc., USA). The 16S rRNA V3-V4 region, obtained from high-quality DNA, was then targeted for amplicon library preparation using Illumina primer sets and a Nextera XT index kit (Illumina, Inc.), per the 16S metagenomic sequencing library preparation protocol. Amplicon libraries with a mean fragment size of 576 bp were sequenced on the Illumina MiSeq platform using 2 × 300-bp paired-end chemistry; 476,528 of the generated paired-end raw reads were trimmed using Trimmomatic version 0.3 (11). QIIME modules were queried against the Greengenes database utilized for operational taxonomic unit (OTU) binning and saved as BIOM files for alpha diversity and rarefaction analysis (12).

OTU binning discovered 4,674 species belonging to 25 major phyla, of which Proteobacteria (33.12%), Actinobacteria (14.58%), Acidobacteria (12.81%), and Bacteroidetes (9.89%) were abundant. Our data provide new insights into the complexity and diversity of microfloral residents of this unique biome. A high OTU discovery rate indicates the existence of highly diverse microbiota among the rich floral and faunal resources in the Western Ghats.

### Accession number(s).

Data for the genome sequence reported here have been submitted to NCBI/GenBank under SRA accession number SRP131503.
